# Investigating Information Geometry in Classical and Quantum Systems through Information Length

**DOI:** 10.3390/e20080574

**Published:** 2018-08-03

**Authors:** Eun-jin Kim

**Affiliations:** School of Mathematics and Statistics, University of Sheffield, Sheffield S3 7RH, UK; e.kim@sheffield.ac.uk or

**Keywords:** stochastic processes, Langevin equation, Fokker–Planck equation, information length, Fisher information, relaxation, chaos, attractor, probability density function

## Abstract

Stochastic processes are ubiquitous in nature and laboratories, and play a major role across traditional disciplinary boundaries. These stochastic processes are described by different variables and are thus very system-specific. In order to elucidate underlying principles governing different phenomena, it is extremely valuable to utilise a mathematical tool that is not specific to a particular system. We provide such a tool based on information geometry by quantifying the similarity and disparity between Probability Density Functions (PDFs) by a metric such that the distance between two PDFs increases with the disparity between them. Specifically, we invoke the information length L(t) to quantify information change associated with a time-dependent PDF that depends on time. L(t) is uniquely defined as a function of time for a given initial condition. We demonstrate the utility of L(t) in understanding information change and attractor structure in classical and quantum systems.

## 1. Introduction

Stochastic processes are ubiquitous in nature and laboratories, and play a major role across traditional disciplinary boundaries. Due to the randomness associated with stochasticity, the evolution of these systems is not deterministic but instead probabilistic. Furthermore, these stochastic processes are described by different variables and are thus very system-specific. This system-specificity makes it impossible to make comparison among different processes. In order to understand universality or underlying principles governing different phenomena, it is extremely valuable to utilise a mathematical tool that is not specific to a particular system. This is especially indispensable given the diversity of stochastic processes and the growing amount of data.

Information geometry provides a powerful methodology to achieve this goal. Specifically, the similarity and disparity between Probability Density Functions (PDFs) is quantified by a metric [1] such that the distance between two PDFs increases with the disparity between them. This was the very idea behind a statistical distance [2] based on the Fisher (or Fisher–Rao) metric [3] which represents the total number of statistically different states between two PDFs in Hilbert space for quantum systems. The analysis in [2] was extended to impure (mixed-state) quantum systems using a density operator by [4]. Other related work includes [5,6,7,8,9,10,11,12]. For Gaussian PDFs, a statistically different state is attained when the physical distance exceeds the resolution set by the uncertainty (PDF width).

This paper presents a method to define such distance for a PDF which changes continuously in time, as is often the case of non-equilibrium systems. Specifically, we invoke the information length L(t) according to the total number of statistically different states that a system evolves through in time. L(t) is uniquely defined as a function of time for a given initial condition. We demonstrate the utility of L(t) in understanding information change and attractor structure in classical and quantum systems [13,14,15,16,17,18,19,20,21].

This paper is structured as follows: Section 2 discusses information length and Section 3 investigates attractor structure. Section 4 and Section 5 present the analysis of classical music and quantum systems, respectively. Conclusions are found in Section 6.

## 2. Information Length

Intuitively, we define the information length L by computing how quickly information changes in time and then measuring the clock time based on that time scale. Specifically, the time-scale of information change τ can be computed by the correlation time of a time-dependent PDF, say p(x,t), as follows.

(1) $$\begin{array}{ccc} \frac{1}{\tau^{2}} & = & \int dx\frac{1}{p(x,t)}{\left[\frac{\partial p(x,t)}{\partial t}\right]}^{2}. \end{array}$$

From Equation (1), we can see that the dimension of τ=τ(t) is time and serves as a dynamical time unit for information change. L(t) is the total information change between time 0 and *t*:

(2) $$\begin{array}{ccc} L(t) & = & \int_{0}^{t}\frac{dt_{1}}{\tau (t_{1})}=\int_{0}^{t}dt_{1}\sqrt{\int dx\frac{1}{p(x,t_{1})}{\left[\frac{\partial p(x,t_{1})}{\partial t_{1}}\right]}^{2}}. \end{array}$$

In principle, τ(t) in Equation (1) can depend on time, so we need the integral for L in Equation (2). To make an analogy, we can consider an oscillator with a period τ=2 s. Then, within the clock time 10 s, there are five oscillations. When the period τ is changing with time, we need an integration of dt/τ over the time interval.

We now recall how τ(t) and L(t) in Equations (1) and (2) are related to the relative entropy (Kullback–Leibler divergence) [15,16]. We consider two nearby PDFs $p_{1}=p\left(x,t_{1}\right)$ and $p_{2}=p\left(x,t_{2}\right)$ at time $t=t_{1}$ and $t_{2}$ and the limit of a very small $\delta t=t_{2}-t_{1}$ to do Taylor expansion of $D\left[p_{1},p_{2}\right]=\int dx\;p_{2}\ln\left(p_{2}/p_{1}\right)$ by using

(3) $$\begin{array}{ccc} \frac{\partial }{\partial t_{1}}D\left[p_{1},p_{2}\right] & = & -\int dx\;p_{2}\frac{\partial_{t_{1}}p_{1}}{p_{1}}, \end{array}$$

(4) $$\begin{array}{ccc} \frac{\partial^{2}}{\partial t_{1}^{2}}D\left[p_{1},p_{2}\right] & = & \int dx\;p_{2}\left\{\frac{{\left(\partial_{t_{1}}p_{1}\right)}^{2}}{p_{1}^{2}}-\frac{\partial_{t_{1}}^{2}p_{1}}{p_{1}}\right\}, \end{array}$$

(5) $$\begin{array}{ccc} \frac{\partial }{\partial t_{2}}D\left[p_{1},p_{2}\right] & = & \int dx\;\left\{\partial_{t_{2}}p_{2}+\partial_{t_{2}}p_{2}\left[\ln p_{2}-\ln p_{1}\right]\right\}, \end{array}$$

(6) $$\begin{array}{ccc} \frac{\partial^{2}}{\partial t_{2}^{2}}D\left[p_{1},p_{2}\right] & = & \int dx\;\left\{\partial_{t_{2}}^{2}p_{2}+\frac{{\left(\partial_{t_{2}}p_{2}\right)}^{2}}{p_{2}}+\partial_{t_{2}}^{2}p_{2}\left[\ln p_{2}-\ln p_{1}\right]\right\}. \end{array}$$

In the limit $t_{2}\to t_{1}=t$ ($p_{2}\to p_{1}=p$), Equations (3)–(6) give us

(7) $$\begin{array}{ccc} \lim_{t_{2}\to t_{1}}\frac{\partial }{\partial t_{1}}D\left[p_{1},p_{2}\right] & = & \lim_{t_{2}\to t_{1}}\frac{\partial }{\partial t_{2}}D\left[p_{1},p_{2}\right]=\int dx\partial_{t}p=0,\\ \lim_{t_{2}\to t_{1}}\frac{\partial^{2}}{\partial t_{1}^{2}}D\left[p_{1},p_{2}\right] & = & \lim_{t_{2}\to t_{1}}\frac{\partial^{2}}{\partial t_{2}^{2}}D\left[p_{1},p_{2}\right]=\int dx\frac{{\left(\partial_{t}p\right)}^{2}}{p}=\frac{1}{\tau^{2}}. \end{array}$$

Up to $O({\left(dt\right)}^{2})$ ($dt=t_{2}-t_{1}$), Equation (7) and $D(p_{1},p_{1})=0$ lead to

(8) $$D\left[p_{1},p_{2}\right]=\frac{1}{2}\left[\int dx\frac{{\left(\partial_{t}p\left(x,t\right)\right)}^{2}}{p(x,t)}\right]{\left(dt\right)}^{2},$$

and thus the infinitesimal distance $dl(t_{1})$ between $t_{1}$ and $t_{1}+dt$ as

(9) $$dl\left(t_{1}\right)=\sqrt{D[p_{1},p_{2}]}=\frac{1}{\sqrt{2}}\sqrt{\int dx\frac{{\left(\partial_{t_{1}}p\left(x,t_{1}\right)\right)}^{2}}{p(x,t_{1})}}dt.$$

By summing $dt(t_{i})$ for i=0,1,2,…,n−1 (where n=t/dt) in the limit dt→0, we have

(10) $$\lim_{dt\to 0}\sum_{i=0}^{n-1}dl\left(idt\right)=\lim_{dt\to 0}\sum_{i=0}^{n-1}\sqrt{D[p(x,idt),p(x,(i+1)]}\;dt\propto \int_{0}^{t}dt_{1}\;\sqrt{\int dx\frac{{\left(\partial_{t_{1}}p\left(x,t_{1}\right)\right)}^{2}}{p(x,t_{1})}}=L\left(t\right),$$

where L(t) is the information length. Thus, L is related to the sum of infinitesimal relative entropy. It cannot be overemphasised that L is a Lagrangian distance between PDFs at time 0 and *t* and sensitively depends on the particular path that a system passed through reaching the final state. In contrast, the relative entropy D[p(x,0),p(x,t)] depends only on PDFs at time 0 and *t* and thus does not tell us about intermediate states between initial and final states.

## 3. Attractor Structure

Since L(t) represents the accumulated change in information (due to the change in PDF) at time *t*, L(t) settles to a constant value $L_{\infty }$ when a PDF reaches its final equilibrium PDF. The smaller $L_{\infty }$, the smaller number of states that the initial PDF passes through to reach the final equilibrium. Therefore, $L_{\infty }$ provides us with a unique representation of a path-dependent, Lagrangian measure of the distance between a given initial and final PDF. We will utilise this property to map out the attractor structure by considering a narrow initial PDF at a different peak position $y_{0}$ and by measuring $L_{\infty }$ against $y_{0}$. We are particularly interested in how the behaviour of $L_{\infty }$ against $y_{0}$ depends on whether a system has a stable equilibrium point or is chaotic.

### 3.1. Linear vs. Cubic Forces

We first consider the case where a system has a stable equilibrium point when there is no stochastic noise and investigate how $L_{\infty }$ is affected by different deterministic forces [15,16]. We consider the following Langevin equation [22] for a variable *x*:

(11) $$\frac{dx}{dt}=F\left(x\right)+\xi .$$

Here, ξ is a short (delta) correlated stochastic noise with the strength *D* as

(12) $$\langle \xi \left(t\right)\xi \left(t^{\prime }\right)\rangle =2D\delta \left(t-t^{\prime }\right),$$

where the angular brackets denote the average over ξ and 〈ξ〉=0. We consider two types of *F*, which both have a stable equilibrium point x=0; the first one is the linear force F=−γx (γ>0 is the frictional constant) which is the familiar Ornstein–Uhlenbeck (O-U) process, a popular model for a noisy relaxation system (e.g., [23]). The second is the cubic force $F=-\mu x^{3}$ where μ represents the frictional constant. Note that, in these models, the dimensions of γ ($s^{-1}$) and μ ($s^{-1}m^{-2}$) are different.

Equivalent to the Langevin equation governed by Equations (11) and (12) is the Fokker–Planck equation [22]

(13) $$\frac{\partial }{\partial t}p\left(x,t\right)=\frac{\partial }{\partial x}\left\{-F\left(x\right)+D\frac{\partial }{\partial x}\right\}p\left(x,t\right).$$

As an initial PDF, we consider a Gaussian PDF

(14) $$p\left(x_{0},0\right)=\sqrt{\frac{\beta_{0}}{\pi }}e^{-\beta_{0}{\left(x_{0}-y_{0}\right)}^{2}}.$$

Then, for the O-U process, the PDF remains Gaussian for all time with the following form [15,16]:

(15) $$p\left(x,t\right)=\sqrt{\frac{\beta (t)}{\pi }}e^{-\beta \left(t\right){\left(x-\langle x\rangle \right)}^{2}}.$$

In Equations (14) and (15), $\langle x\rangle =y_{0}e^{-\gamma t}$ is the mean position and $y_{0}$ is its initial value; $\beta_{0}$ is the inverse temperature at t=0, which is related to the variance at t=0 as $\langle {\left(x_{0}-y_{0}\right)}^{2}\rangle =\frac{1}{2\beta_{0}}=\frac{D_{0}}{\gamma }$. The fluctuations level (variance) changes with time, with time-dependent β(t) given by

(16) $$\langle {\left(x-\langle x\rangle \right)}^{2}\rangle =\frac{1}{2\beta (t)}=\frac{D(1-e^{-2\gamma t})}{\gamma }+\frac{e^{-2\gamma t}}{2\beta_{0}}.$$

Note that, when $D=D_{0}$, $\beta \left(t\right)=\beta_{0}=\frac{\gamma }{2D}$ for all *t*, PDF maintains the same width for all *t*.

For this Gaussian process, β and 〈x〉 constitute a parameter space on which the distance is defined with the Fisher metric tensor [3] $g_{ij}$ (i,j=1,2) as [16]

(17) $$\begin{array}{c} g_{ij}=\int dx\frac{1}{p(x,t)}\frac{\partial p}{\partial z^{i}}\frac{\partial p}{\partial z^{j}}=\left(\begin{array}{cc} \frac{1}{2\beta^{2}} & 0\\ 0 & 2\beta^{} \end{array}\right), \end{array}$$

where i,j=1,2, $z^{1}=\beta$, $z^{2}=\langle x\rangle$. This enables us to recast $\frac{1}{\tau^{2}}$ in Equation (1) in terms of $g_{ij}$ as

(18) $$\begin{array}{c} \frac{1}{\tau^{2}}=\frac{1}{2\beta^{2}}{\left(\frac{d\beta }{dt}\right)}^{2}+2\beta {\left(\frac{d\langle x\rangle }{dt}\right)}^{2}\;\;=g_{ij}\frac{dz^{i}}{dt}\frac{dz^{j}}{dt}. \end{array}$$

The derivation of the first relation in Equation (18) is provided in Appendix A (see Equation (A2)). Using Equations (2) and (18), we can calculate L analytically for this O-U process (see also Appendix A).

In comparison, theoretical analysis can be done only in limiting cases such as small and large times for the cubic process [17,24]. In particular, the stationary PDF for large time is readily obtained as

(19) $$p\left(x\right)=\frac{2\beta_{c}^{\frac{1}{4}}}{\Gamma \left(\frac{1}{4}\right)}e^{-\beta_{c}x^{4}},$$

where $\beta_{c}=\frac{\mu }{4D}$. For the exact calculation of L(t), Equation (13) is to be solved numerically.

**Figure 1:**
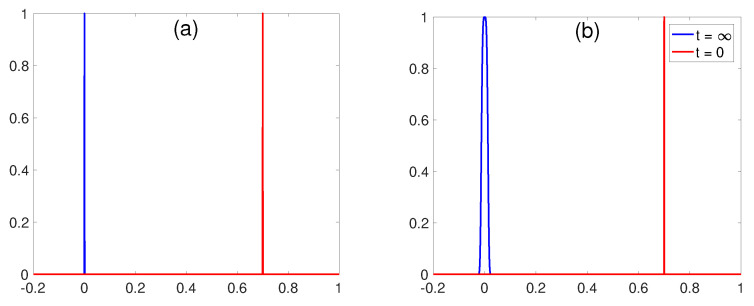
Initial (red) and final (blue) Probability Density Functions (PDFs) for the O-U process in (**a**) and the cubic process in (**b**).

To summarise, due to the restoring forcing *F*, the equilibrium is given by a PDF around x=0, Gaussian for linear force and quartic exponential for cubic force. If we were to pick any point in *x*, say $y_{0}$, we are curious about how close $y_{0}$ is to the equilibrium and how F(x) affects it. To determine this, we make a narrow PDF around $x=y_{0}$ (see Figure 1) at t=0 and measure $L_{\infty }$. The question is how this $L_{\infty }$ depends on $y_{0}$. We repeat the same procedure for the cubic process, as shown in Figure 1, and examine how $L_{\infty }$ depends on $y_{0}$.

**Figure 2:**
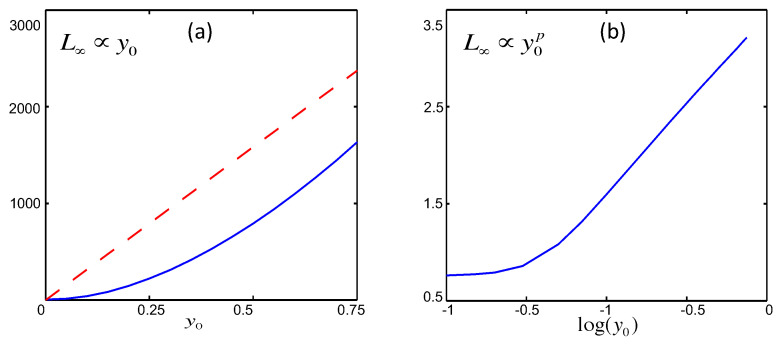
(**a**): $L_{\infty }$ against $\langle x\left(t=0\right)\rangle =y_{0}$ for the linear process in red dashed line and for the cubic process in blue solid line; (**b**): $L_{\infty }$ against $\langle x\left(t=0\right)\rangle =y_{0}$ for the cubic process on log-log scale (data from [17]).

$L_{\infty }$ as a function of $y_{0}$ is shown for both linear (in red dotted line) and cubic (in blue solid line) processes in Figure 2. In the linear case we can see a clear linear relation between $y_{0}$ and $L_{\infty }$, meaning that the information length preserves the linearity of the system. This linear relationship holds for all *D* and $D_{0}$. In particular, when $D=D_{0}$, we can show that $L_{\infty }=\frac{1}{\sqrt{D/\gamma }}y_{0}$ by taking the limit of t→∞ (y→0) in Equation (A10).

In contrast, for the cubic process, the relation is not linear, and the log-log plot on the right in Figure 2 shows a power-law dependence with the power-law index *p*. This power-law index *p* varies between 1.52 and 1.91 and depends on the width ($\propto D_{0}^{1/2}$) of initial PDF and stochastic forcing amplitude *D*, as shown in [16]. This indicates that nonlinear force breaks the linear scaling of geometric structure and changes it to power-law scalings. In either cases here, $L_{\infty }$ has a smooth variation with $y_{0}$ with its minimum value at $y_{0}=0$ since the equilibrium point 0 is stable. This will be compared with the behaviour in chaotic systems in Section 3.2.

### 3.2. Chaotic Attractor

Section 3.1 demonstrates that the minimum value of $L_{\infty }$ occurs at a stable equilibrium point [15,16]. We now show that in contrast, in the case of a chaotic attractor, the minimum value of $L_{\infty }$ occurs at an unstable point [13]. To this end, we consider a chaotic attractor using a logistic map [13]. The latter is simply given by a rule as to how to update the value *x* at t+1 from its previous value at *t* as follows [25]

(20) $$x_{t+1}=1-ax_{t}^{2},$$

where x=[−1,1] and *a* is a parameter, which controls the stability of the system.

**Figure 3:**
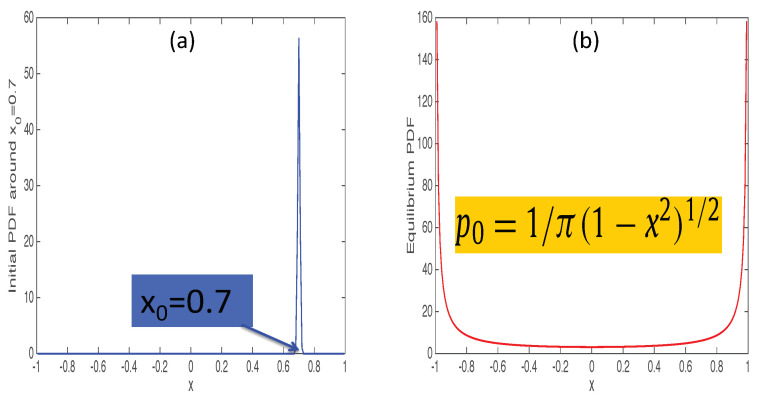
(**a**): an initial narrow PDF at the peak $x_{0}=0.7$; (**b**): the invariant density of a logistic map.

**Figure 4:**
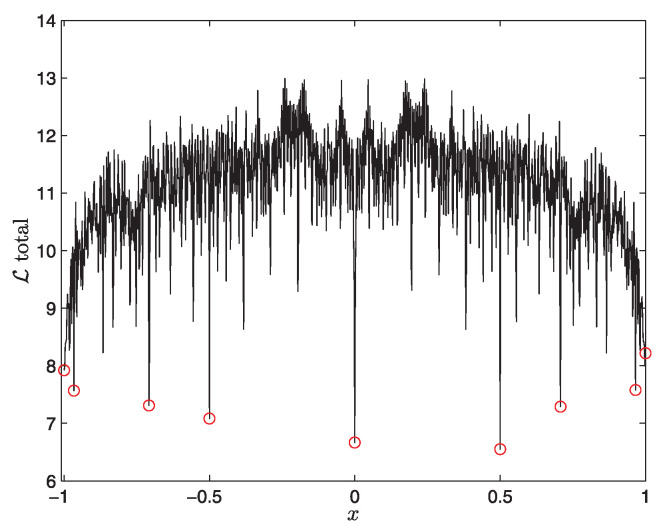
$L_{\infty }$ against the peak position $x=x_{0}$ of an initial PDF in the chaotic regime of a logistic map (Reprinted from *Physics Letters A*, 379, S.B. Nicholson & E. Kim, Investigation of the statistical distance to reach stationary distributions, 83-88, Copyright (2015), with permission from Elsevier).

As we are interested in a chaotic attractor, we chose the value a=2 so that any initial value $x_{0}$ evolves to a chaotic attractor given by an invariant density (shown in the right panel of Figure 3). A key question is then whether all values of $x_{0}$ are similar as they all evolve to the same invariant density in the long time limit. To address how close a particular point $x_{0}$ is to equilibrium, we (i) consider a narrow initial PDF around $x_{0}$ at t=0, (ii) evolve it until it reaches the equilibrium distribution, (iii) measure the $L_{\infty }$ between initial and final PDF, and (iv) repeat steps (i)–(iii) for many different values $x_{0}$. For example, for $x_{0}=0.7$, the initial PDF is shown on the left and final PDF on the right in Figure 3. We show $L_{\infty }$ against $x_{0}$ in Figure 4. A striking feature of Figure 4 is an abrupt change in $L_{\infty }$ for a small change in $x_{0}$. This means that the distance between $x_{0}$ and the final chaotic attractor depends sensitively on $x_{0}$. This sensitive dependence of $L_{\infty }$ on x(t=0) means that a small change in the initial condition $x_{0}$ causes a large difference in a path that a system evolves through and thus $L_{\infty }$. This is a good illustration of a chaotic equilibrium and is quite similar to the sensitive dependence of the Lyapunov exponent on the initial condition [25]. That is, our $L_{\infty }$ provides a new methodology to test chaos. Another interesting feature of Figure 4 are several points with small values of $L_{\infty }$, shown by red circles. In particular, $x_{0}=0.5$ has the smallest value of $L_{\infty }$, indicating that the unstable point is closest to the chaotic attractor. That is, an unstable point is most similar to the chaotic attractor and thus minimises $L_{\infty }$.

## 4. Music: Can We See the Music?

Our methodology is not system-specific and applicable to any stochastic processes. In particular, given any time-dependent PDFs that are computed from a theory, simulations or from data, we can compute L(t) to understand information change. As an example, we apply our theory to music data and discuss information change associated with different pieces of classical music. In particular, we are interested in understanding differences among famous classical music in view of information change. To gain an insight, we used the MIDI file [26], computed time-dependent PDFs and the information length as a function of time [14].

**Figure 5:**
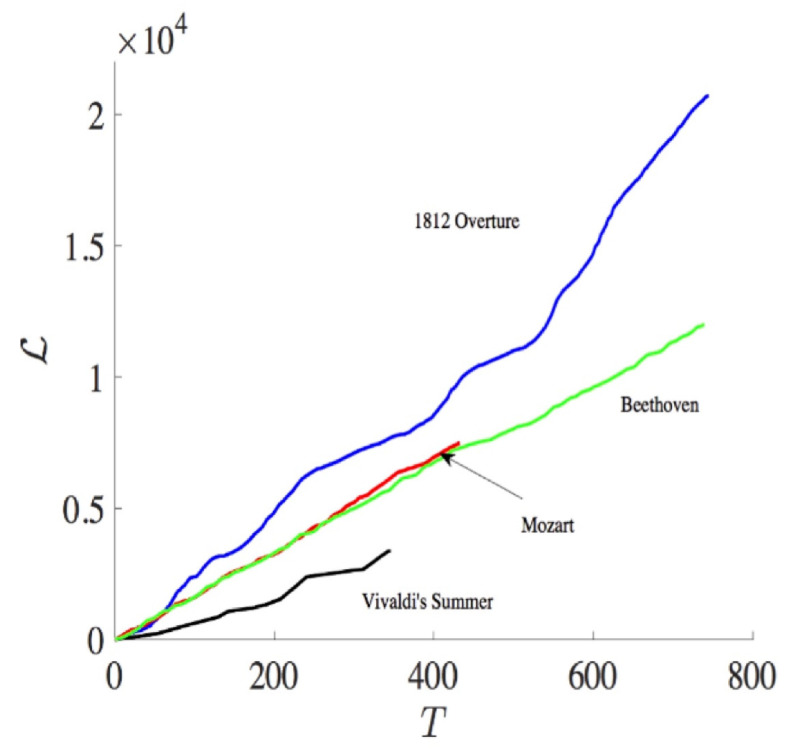
L(t) against time *T* for different composers (from [14]).

**Figure 6:**
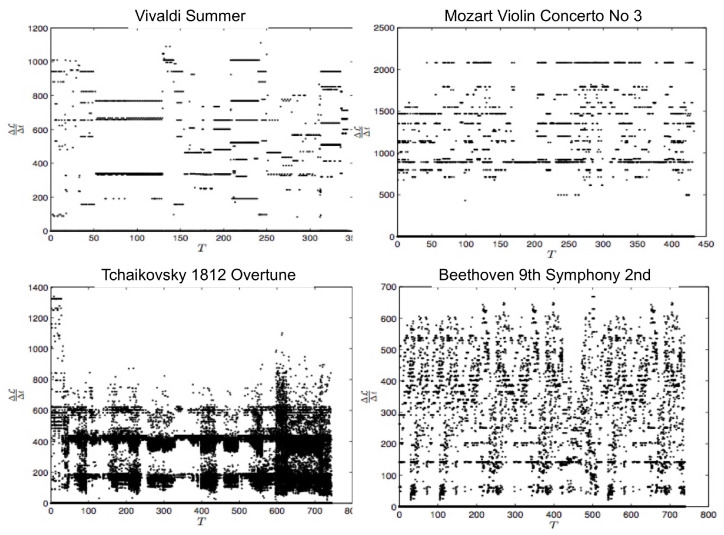
$\frac{1}{\tau }=\frac{dL}{dt}$ for different composers shown in Figure 5 (from [14]).

Specifically, the midi file stores a music by the MIDI number according to 12 different music notes (C, C${}^{\#}$, D, D${}^{\#}$, E, F, F${}^{\#}$, G, G${}^{\#}$, A, A${}^{\#}$, B) and 11 different octaves, with the typical time Δt between the two adjacent notes of order $\Delta t∼10^{-3}$ s. In order to construct a PDF, we specify 129 statistically different states according to the MIDI number and one extra rest state (see Table 1 in [14]) and calculate an instantaneous PDF (see Figure S1 in [14]) from an orchestra music by measuring the frequency (the total number of times) that a particular state is played by all instruments at a given time. Thus, the time-dependent PDFs are defined in discrete time steps with $\Delta t∼10^{-3}$, and the discrete version of L (Equation (7) in [14]) is used in numerical computation. Figure 5 shows L(t) against time for Vivaldi’s Summer, Mozart, Tchaikovsky’s 1812 Overture, and Beethoven’s Ninth Symphony 2nd movement. We observe the difference among different composers, in particular, more classical, more subtle in information change. We then look at the rate of information change against time for different music by calculating the gradient of L ($\frac{dL}{dt}=1/\tau$) in Figure 6, which also manifests the most subtle change in information length for Vivaldi and Mozart.

## 5. Quantum Systems

Finally, we examine quantum effects on information length [21]. In Quantum Mechanics (QM), the uncertainty relation $\Delta x\Delta P\geq \frac{\hbar }{2}$ between position *x* and momentum *P* gives us an effect quite similar to a stochastic noise. We note here that we are using *P* to denote the momentum to distinguish it from a PDF (p(x,t)). For instance, the trajectory of a particle in the x−P phase space is random and not smooth. Furthermore, the phase volume *h* plays the role of resolution in the phase space, one unit of information given by the phase volume *h*. Thus, the total number of states is given by the total phase volume divided by *h*. This observation points out a potentially different role of the width of PDF in QM in comparison with the classical system since a wider PDF in QM occupies a larger region of *x* in the phase space, with the possibility of increasing the information.

To investigate this, for simplicity, we consider a particle of mass *m* under a constant force *F* and assume an initial Gaussian wave function around $x^{\prime }=0$ [21]

(21) $$\psi \left(x^{\prime },0\right)={\left(\frac{2\beta_{0}}{\pi }\right)}^{\frac{1}{4}}e^{-\beta_{0}x^{\prime 2}+ik_{0}x^{\prime }},$$

where $k_{0}=P_{0}/\hbar$ is the wave number at t=0, $D_{x}={\left(2\beta_{0}\right)}^{-1/2}$ is the width of the initial wave function, and $P_{0}$ is the initial momentum. A time-dependent PDF p(x,t) is then found as (e.g., see [21,27]):

(22) $$p\left(x,t\right)={\left|\psi \left(x,t\right)\right|}^{2}=\sqrt{\frac{\beta (t)}{\pi }}e^{-\beta \left(t\right){\left(x-\langle x\rangle \right)}^{2}}.$$

Here,

(23) $$\begin{array}{cc} \beta \left(t\right)=\frac{2\beta_{0}m^{2}}{m^{2}+{\left(2\hbar \beta_{0}t\right)}^{2}},\;\;\; & \langle x\rangle =\frac{\hbar k_{0}t}{m}+\frac{Ft^{2}}{2m}. \end{array}$$

Equation (22) clearly shows that the PDF is Gaussian, with the mean $\langle x\rangle =\frac{\hbar k_{0}t}{m}+\frac{Ft^{2}}{2m}$ and the variance

(24) $$\mathrm{Var}\left(t\right)=\langle {\left(x-\langle x\rangle \right)}^{2}\rangle =\frac{1}{4\beta }=\frac{1}{4\beta_{0}}+\frac{\beta_{0}\hbar^{2}t^{2}}{m^{2}}=\mathrm{Var}\left(0\right)+\frac{\hbar^{2}t^{2}}{4\mathrm{Var}\left(0\right)m^{2}}.$$

In Equation (24), $\mathrm{Var}\left(0\right)=\langle {\left(x\left(0\right)-\langle x\left(0\right)\rangle \right)}^{2}\rangle =\frac{1}{4\beta_{0}}=\frac{D_{x}}{2}$ is the initial variance. We note that the last term in Equation (24) increases quadratically with time *t* due to the quantum effect, the width of wave function becoming larger over time. Obviously, this effect vanishes as ℏ→0.

Since the PDF in Equation (22) is Gaussian, we can use Equation (18) to find (e.g., see [16])

(25) $$\begin{array}{ccc} \frac{1}{\tau^{2}} & = & 2t^{2}\frac{1}{{\left(T^{2}+t^{2}\right)}^{2}}+2\beta_{0}\frac{T^{2}}{T^{2}+t^{2}}v_{0}^{2}{\left[1+\frac{Ft}{\hbar k_{0}}\right]}^{2}, \end{array}$$

where $T=\frac{m}{2\hbar \beta_{0}}$, the time scale of the broadening of the initial wave function [21]. It is interesting to note that when there is no external constant force *F*, the two terms in Equation (25) decrease for large time *t*, making τ large. The situation changes dramatically in the presence of *F* in Equation (25) as the second term approaches a constant value for large time. The region with the same value of τ signifies that the rate of change in information is constant in time, and was argued to be an optimal path to minimise the irreversible dissipation (e.g., [16]). Physically, this geodesic arises when when the broadening of a PDF is compensated by momentum Ft which increases with time. Mathematically, the limit t→∞ reduces Equation (25) and thus L to

(26) $$\frac{1}{\tau }∼\frac{FD_{x}}{\hbar },\;\;\;L∼\frac{\left(Ft\right)D_{x}}{\hbar }.$$

Since Ft=P and $D_{x}={\left(2\beta_{0}\right)}^{-1/2}$ is the width of the wave function at t=0, $FtD_{x}$ in Equation (26) represents the volume in the P−x phase space spanned by this wave function. This reflects the information changes associated with the coverage of a phase volume *ℏ*. Interestingly, similar results are also obtained in the momentum representation where L is computed from the PDF p(P,t) in the momentum space:

(27) $$\begin{array}{ccc} p\left(P,t\right)=\sqrt{\frac{\lambda }{\pi }}e^{-\lambda {\left(p-(mv_{0}+Ft)\right)}^{2}}, & \frac{1}{\tau^{2}}=2\lambda F^{2}, & L=\sqrt{2\lambda }Ft, \end{array}$$

where $\lambda =\frac{1}{2\hbar^{2}\beta_{0}}$. In Equation (27), τ is obviously constant, and L linearly increases with time *t*. We can see even a strong similarity between Equation (27) and Equation (26) as t→∞ once using $L\propto \sqrt{2\lambda }Ft∼\left(Ft\right)D_{x}/\hbar$. In view of the complementary relation between position and momentum in quantum systems, the similar result for L in momentum and position space highlights the robustness of the geodesic.

## 6. Conclusions

We investigated information geometry associated with stochastic processes in classical and quantum systems. Specifically, we introduced τ(t) as a dynamical time scale quantifying information change and calculated L(t) by measuring the total clock time *t* by τ. As a unique Lagrangian measure of the information change, $L_{\infty }$ was demonstrated to be a novel diagnostic for mapping out an attractor structure. In particular, $L_{\infty }$ was shown to capture the effect of different deterministic forces through the scaling of $L_{\infty }$ again the peak position of a narrow initial PDF. For a stable equilibrium, the minimum value of $L_{\infty }$ occurs at the equilibrium point. In comparison, in the case of a chaotic attractor, $L_{\infty }$ exhibits a sensitive dependence on initial conditions like a Lyapunov exponent. We then showed the application of our method to characterize the information change associated with classical music (e.g., see [14]). Finally, we elucidated the effect of the width of a PDF on information length in quantum systems. Extension of this work to impure (mixed-state) quantum systems and investigation of Riemannian geometry on the space of density operators would be of particular interest for future work.

## Appendix A. ℒ for the O-U Process

To make this paper self-contained, we provide here the main steps for the derivation of L for the O-U process [15,16]. We use $y=\langle x\rangle =y_{0}e^{-\gamma t}$ in p(x,t) in Equation (12) and differentiate it to find

(A1) $$\frac{\partial p}{\partial t}=\left[\dot{\beta }\left(\frac{1}{2\beta }-{\left(x-y\right)}^{2}\right)+2\beta \left(x-y\right)\dot{y}\right]p.$$

Equations (A1) and (1) and using the properties of a Gaussian PDF [$\langle {\left(x-y\right)}^{2}\rangle =\frac{1}{2\beta }$, $\langle {\left(x-y\right)}^{4}\rangle =3{\langle {\left(x-y\right)}^{2}\rangle }^{2}$] lead to

(29) $$\begin{array}{ccc} \frac{1}{\tau^{2}} & = & \frac{1}{2\beta {\left(t\right)}^{2}}{\left(\frac{d\beta }{dt}\right)}^{2}+2\beta {\left(\frac{dy}{dt}\right)}^{2}. \end{array}$$

We express β in Equation (16) in terms of $T=2\beta_{0}D\left(e^{2\gamma t}-1\right)+\gamma$ as $\beta =\frac{\gamma \beta_{0}e^{2\gamma t}}{T}.$ Differentiating this and using $r=2\beta_{0}D-\gamma$ then give

(A3) $$\frac{{\dot{\beta }}^{2}}{2\beta^{2}}=2\gamma^{2}r^{2}\frac{1}{T^{2}}.$$

Similarly, using $\frac{dy}{dt}=-\gamma y_{0}e^{-\gamma t}$, $T=2\beta_{0}D\left(e^{2\gamma t}-1\right)+\gamma$ and $q=\beta_{0}\gamma {y_{0}}^{2}$, we obtain

(A4) $$\begin{array}{c} 2\beta {\dot{y}}^{2}=2q\gamma^{2}\frac{1}{T}. \end{array}$$

Using these results, Equations (A3) and (A4) in (A2) gives us

(32) $$\begin{array}{c} \frac{1}{\tau^{2}}=\frac{1}{2\beta^{2}}{\left(\frac{d\beta }{dt}\right)}^{2}+2\beta {\left(\frac{dy}{dt}\right)}^{2}=\frac{2\gamma^{2}}{T^{2}}\left(r^{2}+qT\right). \end{array}$$

Again, in Equation (A5), $q=\beta_{0}\gamma {y_{0}}^{2}$, $r=2\beta_{0}D-\gamma$, and$T=2\beta_{0}D\left(e^{2\gamma t}-1\right)+\gamma$ [15,16,17]. It is worth noting that *q* and *r*, respectively, arise from the difference in mean position at t=0 and t→∞ (i.e., $y_{0}\neq y\left(t\to \infty \right)$) and in PDF width at t=0 and t→∞ (i.e., $D_{0}\neq D$). Thus, the first and second terms in Equation (A5) represent the information change due to the change in PDF width and the movement of the PDF, respectively. Using $D_{0}=\frac{\gamma }{2\beta_{0}}$, we express *r*, *q* and *T* in Equation (A5) as $q=\frac{\gamma^{2}y_{0}^{2}}{2D_{0}},\;\;r=\gamma \left(\frac{D}{D_{0}}-1\right),\;T=\gamma \left[\frac{D}{D_{0}}\left(e^{2\gamma t}-1\right)+1\right].$ Equations (A5) and (2) then give us

(A6) $$L=\frac{1}{\sqrt{2}}\int_{T_{i}}^{T_{f}}\left\{\frac{1}{T}\frac{1}{T+r}\sqrt{r^{2}+qT}\right\}dT,$$

where $T_{i}=T\left(t=0\right)$ and $T_{f}=T\left(t\right)$. To compute Equation (A6) for r≠0, we use $Y=\sqrt{r^{2}+qT}$ and integrate

(A7) $$L=\frac{1}{\sqrt{2}}{\left[\ln\left(\frac{Y-r}{Y+r}\right)\right]}_{Y_{i}}^{Y_{f}}+\frac{\sqrt{2}}{r}H,\;\;H=\int_{Y_{i}}^{Y_{f}}\frac{qr-r^{2}}{Y^{2}+qr-r^{2}}dY,$$

where $Y_{i}=Y\left(t=0\right)$ and $Y_{f}=Y\left(t\right)$. To calculate *H* in Equation (A7), we need to consider the two cases where q≥r or q<r. First, when q≥r, we use the change of the variable $Y=\sqrt{qr-r^{2}}\tan\theta$ to find

(A8) $$H=\sqrt{qr-r^{2}}{\left[\tan^{-1}\left(\frac{Y}{\sqrt{qr-r^{2}}}\right)\right]}_{Y_{i}}^{Y_{f}}.$$

When q<r, we let $Y=\sqrt{r^{2}-qr}\sec\theta$ and find

(A9) $$H=-\frac{\sqrt{r^{2}-qr}}{2}{\left[\ln\left(\frac{Y-\sqrt{r^{2}-qr}}{Y+\sqrt{r^{2}-qr}}\right)\right]}_{Y_{i}}^{Y_{f}}.$$

When $D=D_{0}$ (r=0), $\beta \left(t\right)=\beta_{0}$ for all *t*. Thus, Equation (2) can easily be calculated directly from Equation (A5) with the result

(A10) $$L=\frac{1}{\sqrt{2}}\int_{T_{i}}^{T_{f}}\frac{\sqrt{q}}{T^{\frac{3}{2}}}dT=-\sqrt{2q}{\left[\frac{1}{\sqrt{T}}\right]}_{T_{i}}^{T_{f}}=\frac{1}{\sqrt{D/\gamma }}\left[y_{0}-y\right],$$

where again $y=\langle x\rangle =y_{0}e^{-\gamma t}$.
